# Synthetic biology approaches to improve biocatalyst identification in metagenomic library screening

**DOI:** 10.1111/1751-7915.12146

**Published:** 2014-08-13

**Authors:** María-Eugenia Guazzaroni, Rafael Silva-Rocha, Richard John Ward

**Affiliations:** 1 Departamento de Química, FFCLRP, University of São PauloRibeirão Preto, SP, Brazil; 2 Departamento de Bioquímica e Imunologia, FMRP, University of São PauloRibeirão Preto, SP, Brazil

## Abstract

There is a growing demand for enzymes with improved catalytic performance or tolerance to process-specific parameters, and biotechnology plays a crucial role in the development of biocatalysts for use in industry, agriculture, medicine and energy generation. Metagenomics takes advantage of the wealth of genetic and biochemical diversity present in the genomes of microorganisms found in environmental samples, and provides a set of new technologies directed towards screening for new catalytic activities from environmental samples with potential biotechnology applications. However, biased and low level of expression of heterologous proteins in *Escherichia coli* together with the use of non-optimal cloning vectors for the construction of metagenomic libraries generally results in an extremely low success rate for enzyme identification. The bottleneck arising from inefficient screening of enzymatic activities has been addressed from several perspectives; however, the limitations related to biased expression in heterologous hosts cannot be overcome by using a single approach, but rather requires the synergetic implementation of multiple methodologies. Here, we review some of the principal constraints regarding the discovery of new enzymes in metagenomic libraries and discuss how these might be resolved by using synthetic biology methods.

## Introduction

Biotechnology takes advantage of processes that utilize biological components or living organisms, and plays an increasing role in industry, agriculture, medicine and energy generation. In the industrial context, many manufacturing processes that previously depended strictly on complex (and frequently harmful) chemical reactions have been superseded by much simpler and safer enzyme-based catalysis (Kirk *et al*., 2002). The introduction of biotechnology in industrial processes generates not only a reduction in the final amount and toxicity of effluents, but can also considerably reduce costs (Herrera, 2004). The number of biotechnology applications has expanded in recent years, and this has created a growing demand for biocatalysts with superior performance or tolerance to extreme application-specific conditions (Lorenz *et al*., 2002; Schloss and Handelsman, 2003). This is particularly true in those industries that produce bulk commodities such as detergents (Maurer, 2004). Similarly, fine-chemical industries require multiple biocatalysts in order to perform highly diverse transformations for the production of new compounds (Homann *et al*., 2004).

The use of enzymes in industrial applications has grown considerably, and represents a market of approximately US$4 billion in 2011 (GIA, 2012), and a number of different categories of enzymes have been used in a wide variety of applications (Schoemaker *et al*., 2003). For example, proteases have been used in detergents, in pharmaceutical and chemical synthesis industries to degrade proteins into amino acids (Gupta *et al*., 2002). Lipases hydrolyse fats to fatty acids and glycerol, and are useful for effluent treatment, detergent production and synthesis of fine chemicals among others (Hasan *et al*., 2006). Glycosyl hydrolases (GHs) catalyse the hydrolysis of carbohydrates to sugars and have found applications in many processes in the textile, pulp and paper, and food production industries (Kirk *et al*., 2002). The food industry also takes advantage of amylases, enzymes that hydrolase starch into sugars (Kirk *et al*., 2002). Furthermore, cellulases are not only useful for fuel production but are also applied in food processing, chemical synthesis and textile industries (Bhat, 2000).

**Fig 1:**
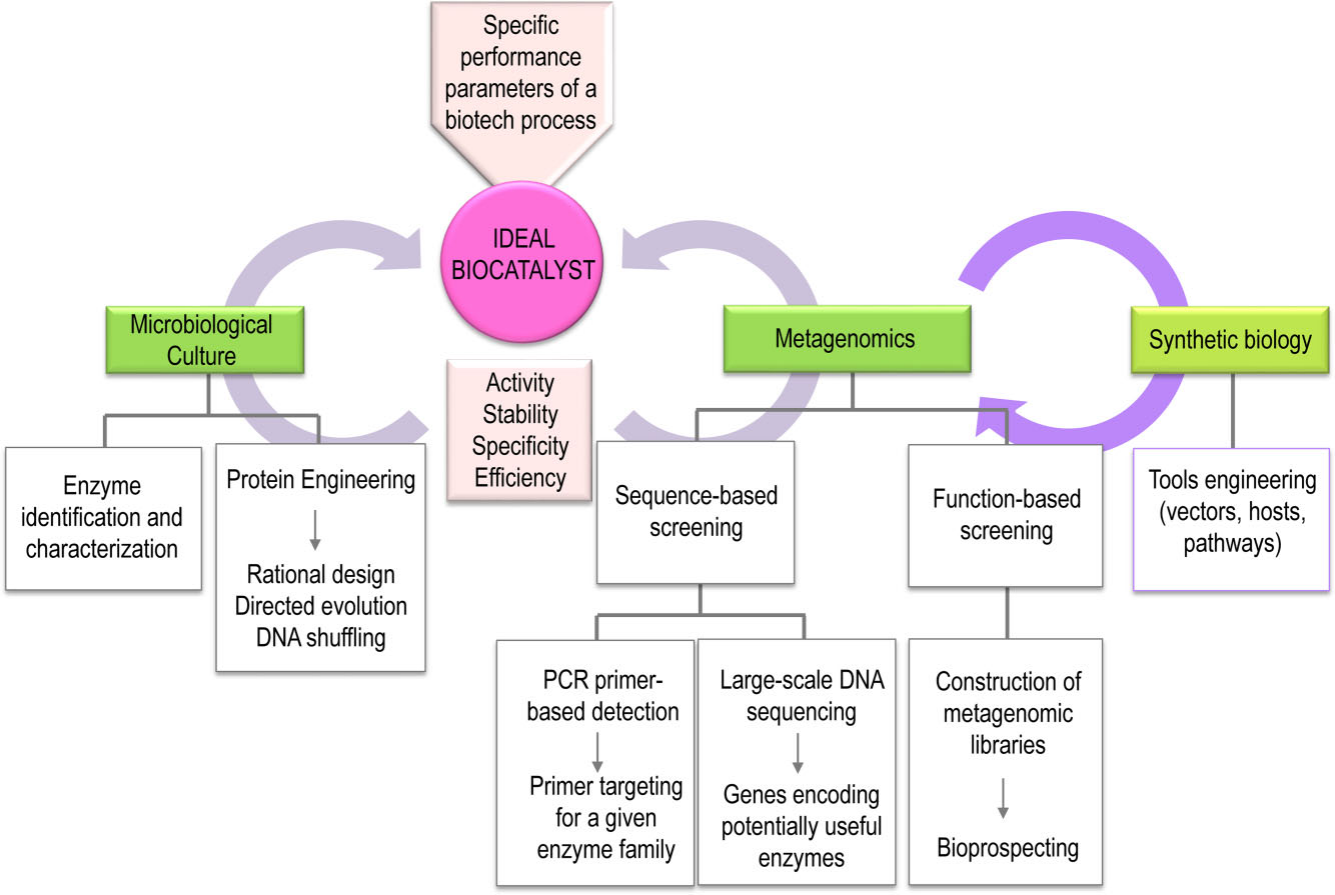
Overall diagram for the identification strategy of an ideal biocatalyst. The identification of enzymes from cultured microorganisms and metagenomics are the two principal approaches currently employed for recovering of genes encoding the desired enzymatic activity for industrial processes. The genes encoding enzymes identified from cultured microorganisms may be cloned and expressed, and parameters such as activity, stability, specificity and efficiency improved using protein rational design and *in vitro* evolution techniques. Metagenomics strategies are based on either activity-based approaches, which involve the construction of expression libraries and its posterior activity screening, or sequence-based approaches. Sequence-based approaches involve either the design of DNA primers for conserved regions of known protein families or data mining of genes encoding potential biocatalysts identified in sequences from next generation sequencing projects. Synthetic biology can provide solutions to the current limitations in activity-based metagenomic approaches. Development of methods for the engineering of new bacterial hosts and molecular biology tools promise to increase the efficiency of discovery of biotechnologically relevant enzymes.

The impact of the use of enzymes in industrial processes has stimulated an increased interest both in identifying new variants with enhanced kinetic parameters and in modifying previously characterized enzymes to increase their suitability for industrial applications (Lorenz *et al*., 2002; Schloss and Handelsman, 2003). Parameters such as activity, efficacy, specificity and stability are used to characterize and select enzymes for different applications (Lorenz and Eck, 2005). Enzymes used in industry have been identified from different sources through a combination of two major strategies: (i) the identification of novel enzymes from cultured microorganisms and (ii) molecular evolution by DNA shuffling and rational design (Lynd *et al*., 2002; Percival Zhang *et al*., 2006; Krogh *et al*., 2010; Ward, 2011; Chen *et al*., 2012) (see Fig. 1). However, enzymes suitable for a given biotechnology application need to work efficiently within specified parameters (Fig. 1), and since those currently used are frequently not optimized, the industrial processes have to be adjusted to accommodate these suboptimal catalysts (Warnecke and Hess, 2009). As a consequence, there is an increasing demand for new biocatalysts with improved properties for industrial applications, such as higher catalytic efficiency on insoluble substrates (as in the case of cellulases used in the production of second generation bioethanol), increased stability at elevated temperature and at defined pH, and higher tolerance to end-product inhibition (Ward, 2011; Singhania *et al*., 2013).

Microorganisms play a central role in biotechnology, not only as tools in molecular biology techniques, but also as the major source of biocatalysts for industrial applications (Fernandez-Arrojo *et al*., 2010). Bearing in mind that the total number of microbial cells on Earth is estimated to be 10^30^ (Turnbaugh and Gordon, 2008), the huge natural wealth regarding protein diversity can be envisaged. Although prokaryotes represent the largest proportion of individual living organisms with an estimated 10^3^–10^5^ microbial species in 1 g of soil (Schloss and Handelsman, 2006), less than 1% can be cultured using existing methodologies (Sleator *et al*., 2008). If we assume that a single genome encodes 4000 proteins (as is the case for the typical bacteria *Escherichia coli*), then 4 × 10^8^ potential proteins might be expected in just 1 g of soil. Supposing that 40% of these proteins display catalytic activity (Dinsdale *et al*., 2008), we might expect to find 1.6 × 10^8^ biocatalysts, which highlights the vast inventory of biological functions available in nature. Metagenomics avoids the necessity of isolation and laboratory cultivation of individual microorganisms, and has become a powerful tool for accessing and exploring the biological and molecular biodiversity present in different natural environments. Over the past decades, many studies using metagenomic approaches have proven to be successful for the recovery of novel enzymes with potential use in industrial applications (Lorenz and Eck, 2005; Fernandez-Arrojo *et al*., 2010). Despite these successes, metagenomic strategies typically have low rates of target identification, and a number of issues need to be addressed in order to improve the screening efficiency of metagenomic libraries. The limits include: (i) bias imposed by host organism expression; (ii) vector performance in particular hosts; and (iii) suitable screening strategies in relation to the specific properties required in the target enzymes. As has already been demonstrated in several recent studies, all these issues may addressed using a synthetic biology approach (Williamson *et al*., 2005; Uchiyama and Watanabe, 2008; Uchiyama and Miyazaki, 2010). In addition to the application of existing synthetic biology approaches, the development of new methodologies is imperative for the next generation of metagenomic studies that aim to recover ideal biocatalysts for given industrial processes. This review focuses on how the interplay between synthetic biology and functional metagenomics can yield novel strategies to obtain ideal candidate enzymes with specific characteristics. We also discuss how innovative synthetic biology applications could help relieve current limitations on metagenomic screening.

## Bioprospecting metagenomes to identify new biocatalysts

### Recent advances and major limitations

Although most environmental bacteria are refractory to cultivation, the biotechnological potential of the uncultivated bacteria can be realized by directly cloning the DNA retrieved from the microbial community (Guazzaroni *et al*., 2010a). The construction and subsequent screening of metagenomic libraries allows identification of the targeted genes encoding the desired catalytic activities (Fig. 1). Accordingly, a well-planned strategy should take into consideration the vector to be used, the host organism for transformation and the screening strategy in order to maximize the rate of identification of the target activities. For example, if single genes or small operons are of interest, the best option is to use a small-insert metagenomic library instead of a large-insert library (Guazzaroni *et al*., 2010a). Small-insert expression libraries, especially those using lambda phage vectors and plasmids, are especially suitable for activity-based screening. The small size of the cloned fragments (up to 8 kb) means that most genes that are present in the appropriate orientation will be under the influence of strong vector promoters, and thus have a good chance of being expressed and detected in activity screens (Ferrer *et al*., 2009). On the other hand, if the goal is biosynthetic pathway mining or functional expression of large multi-enzyme assemblies (for example, in the case of polyketide synthases clusters), the preferred option is library construction using cosmids or fosmid, which can harbour DNA inserts of up to 40 kb in size (Guazzaroni *et al*., 2010a). Since fosmids use both the F-plasmid origin of replication and the partitioning mechanisms present in *E. coli* (Kim *et al*., 1992), the use of these vectors are limited to this host organism. It is noteworthy that the bacterium *E. coli* is the most commonly used host strain for library screening, since many of the currently available genetic tools have been developed in this organism. One example is the use of modified phages that undergo the lytic cycle under controlled conditions, which allows the identification of proteins that would otherwise be toxic for the cell (Guazzaroni *et al*., 2010a). Furthermore, a number of protocols for obtaining high rates of efficiency of transformation in *E. coli* are well established. In addition, kits that facilitate library construction and efficient transformation with different vectors are commercially available (Guazzaroni *et al*., 2010a). However, it is important to appreciate that significant differences in expression modes exist between different taxonomic groups of prokaryotes, and that only 40% of enzymatic activities may be detected by random cloning in *E. coli* (Gabor *et al*., 2004). Therefore, it is likely that also performing metagenomic library screening in hosts other than *E. coli* will expand the range of detectable activities, although achieving this goal will require further optimization of the conditions for high transformation efficiency. Indeed, promising results of metagenomic library screening have been reported in *Streptomyces* spp. (Wang *et al*., 2000), *Rhizobium leguminosarum* (Wexler *et al*., 2005) and diverse Proteobacteria (Craig *et al*., 2010). For example, Wexler and collaborators constructed a library in the broad host-range cosmid pLAFR3 using metagenomic DNA obtained from the microbial community of an anaerobic digester in a wastewater treatment plant (Wexler *et al*., 2005). After screening the metagenomic libraries in *R. leguminosarum*, a single cosmid that enabled *R. leguminosarum* to grow on ethanol as the sole carbon and energy source was recovered. Further analysis identified the presence of a gene encoding an atypical alcohol dehydrogenase that did not confer ethanol utilization ability to either *E. coli* or to *Pseudomonas aeruginosa*, even though the gene was transcribed in both these hosts (Wexler *et al*., 2005). These results show that the use of broad host-range vectors enhances the flexibility of metagenomic library screening. Furthermore, a recent functional metagenomic study showed that recovery of genes conferring acid resistance to *E. coli*, and the subsequent transfer of some of these genes to *P. putida* and *B. subtilis*, expanded the capabilities of these two bacteria to survive harsh acid conditions (Guazzaroni *et al*., 2013). However, in agreement with previous studies (Craig *et al*., 2010), variable gene doses were present due to the use of different cloning plasmids in each host organism, and no quantitative comparison could be made regarding gene expression or activity levels between the hosts. Thus, the developing of robust broad-host-range vectors capable of replication in several different hosts is one of the major challenges in metagenomics, and one to which synthetic biology may make a significant contribution.

In addition to viable library construction, an additional bottleneck in metagenomic screening is related to the low frequency of positive clones that are typically recovered (Vieites *et al*., 2009). Common screening methods are based on the degradation of specific enzyme substrates that result either in the appearance of halos surrounding the positive clones or alternatively on the use of chromogenic substrates (Guazzaroni *et al*., 2010b). Depending on the type of substrate used and on the enzyme screened, detection of positive clones can be assayed directly in solid or in liquid media (Guazzaroni *et al*., 2010b), and the choice of medium will have consequences on the throughput level of the screening method. Similarly, adequate selection of specific activity-driven substrates plays a key role in the success of recovering of the desired enzymatic activity and decreasing the frequency of false positives.

Although methods are available to the direct screening of many enzymes, there is an increasing need to expand the tools available for enzyme detection. An alternative approach to this problem using synthetic biology is the design and implementation of *in vivo* biosensors capable of generate a detectable output in response to the degradation or production of a particular metabolite (Galvao and de Lorenzo, 2006). In this context, substrate-induced gene expression and product-induced gene expression approaches have been developed and successfully applied to the detection of enzymatic activities associated with the metabolic modification of compounds of interest (Uchiyama *et al*., 2005; Uchiyama and Miyazaki, 2010). In a broader approach, the concept of genetic traps has been used to guide the construction of synthetic circuits containing engineered regulators to control gene expression responses to metabolites generated by enzymatic activities present in cloned metagenomic fragments (Uchiyama and Watanabe, 2008). In general, these strategies are particularly useful for screening of libraries where processing of small molecules is targeted (Williamson *et al*., 2005).

### Activity-based versus sequence-based screening strategies

There are two distinct strategies in functional metagenomics for the recovery of sequences encoding the desired enzymatic activity (Fig. 1). First, activity-based approaches involve construction of small- to large-insert expression libraries that are suitable for direct activity screening, such as lambda phage, plasmid, cosmid or copy-controlled fosmid vectors (Lorenz and Eck, 2005). Once a library has been constructed, a critical step is the screening of a large number of clones, and in the case of activity-based screening, thousands of clones may be analysed in a single screen, with the advantage that sequence information is not required. Therefore, this is the strategy that has the potential to identify entirely novel classes of genes encoding known or novel functions (Handelsman, 2004; Daniel, 2005; Gloux *et al*., 2010; Bhat *et al*., 2013). Furthermore, activity-driven screening strategies can potentially provide a means to reveal undiscovered genes or gene families that cannot be detected by sequence-driven approaches.

The most widely used activity-driven screening approach is phenotype detection of the desired activity using chemical dyes and insoluble or chromophore-bearing derivatives of enzyme substrates. When incorporated into the growth medium, these compounds allow the detection of specific catalytic capabilities of individual clones (Ferrer *et al*., 2009). In an elegant example using this approach, the diversity of rumen enzymes was characterized by screening for hydrolase activity in a metagenomic phage library from the rumen content of a dairy cow (Ferrer *et al*., 2005b). In total, 22 clones with distinct hydrolytic activities were identified and characterized, among which four hydrolases exhibited no sequence similarity to enzymes deposited in the public databases, and the putative catalytic residues in the sequences from four other clones showing esterase and cellulase-like activities did not match any known enzymes. Similarly, activity-based screening of metagenomic fosmid libraries from cellulose-depleting microbial communities found in the fresh casts of two different earthworm species retrieved 10 carbohydrate-modifying enzymes (Beloqui *et al*., 2010). Interestingly, two of the GHs identified represented two novel families of β-galactosidases/α-arabinopyranosidases (Beloqui *et al*., 2010). In another example, metagenomic DNA extracted from activated sludge from industrial wastewater was cloned into fosmids and the resulting *E. coli* library was screened for extradiol dioxygenase activity using catechol as the substrate (Suenaga *et al*., 2007). Analysis of the sequences from a total of 38 clones identified various clusters encoding putative metabolic pathways that were dissimilar to previously reported pathways for degradation of the compound (Suenaga *et al*., 2009a,b). Additionally, several other recent examples have shown that the phenotype detection approach can be successfully applied to the identification of novel targeted enzymes such as dehalogenases (Sharma *et al*., 2013), *meta*-cleavage product hydrolases (Alcaide *et al*., 2013), GHs (Ferrer *et al*., 2012; Ko *et al*., 2013), xylanases (Gong *et al*., 2013b) carboxyl esterases and lipases (Martinez-Martinez *et al*., 2013; 2014).

An alternative activity-driven screening approach is based on heterologous complementation of host strains that require the targeted genes for growth under selective conditions. In this strategy, functional complementation permits growth of a clone transformed with a metagenomic DNA insert containing the necessary genes for survival (Wenzel and Muller, 2005). This technique allows the straightforward and rapid screening of complex metagenomic libraries comprising millions of clones, and since false positives are rare, this approach is highly selective for the targeted genes (Simon *et al*., 2009). However, this strategy is limited to the detection of enzymes that catalyse the synthesis of an essential product and for which an auxotrophic host is either available or can readily be constructed. Several examples in the literature have shown that this approach has been successful in the detection of different enzymes such as racemases (Chen *et al*., 2010), DNA polymerases (Simon *et al*., 2009), β-lactamases (Riesenfeld *et al*., 2004), alcohol dehydrogenases (Wexler *et al*., 2005) and enzymes involved in poly-3-hydroxybutyrate metabolism (Wang *et al*., 2006).

Sequence-based approaches have also led to the effective identification of genes encoding enzymes, such as dimethylsulfoniopropionate-degrading enzymes (Varaljay *et al*., 2010), glycerol dehydratases (Knietsch *et al*., 2003), dioxygenases (Morimoto and Fujii, 2009; Sul *et al*., 2009; Zaprasis *et al*., 2010), nitrite reductases (Bartossek *et al*., 2010), hydrazine oxidoreductases (Li *et al*., 2010), chitinases (Hjort *et al*., 2010), glycoside hydrolases (Lee *et al*., 2013), nitrilases (Gong *et al*., 2013a), prephenate dehydrogenases (Jiang *et al*., 2013) and hemicellulases (Yan *et al*., 2013). The application of sequence-based approaches involves the design of polymerase chain reaction (PCR) primers for the target sequences that are derived from conserved regions of known protein families, and this dependence on prior knowledge limits the possibility for identifying new protein families (Ferrer *et al*., 2009). The large-scale sequencing of bulk DNA or metagenomic libraries through deep sequencing techniques provides the raw data for mining sequences encoding potentially useful enzymes. Since homology-based methods are effective only when the information regarding the reference sequences is accurate, a further disadvantage of this approach is its reliance both on existing genome annotations and on the quality and completeness of current databases (Hallin *et al*., 2008). It is worth a cautionary note, since a significant number of genomes in the current databases contain misannotations (Schnoes *et al*., 2009). Considering that the classification of protein families is based on amino acid similarity, novel enzyme families could not be detected by database searching with sequences from metagenomic sequencing or PCR-based detection methods, and might be annotated as hypothetical proteins. A review of prokaryotic protein diversity in different shotgun metagenome studies indicated that 30–60% of the proteins could not be assigned known functions using current public databases (Vieites *et al*., 2009). The advantage of using activity-based rather than sequence-based screening was highlighted in a recent report in which a novel cold-tolerant esterase with low sequence similarity (< 29% amino acid identity) was identified by functional screening of an Antarctic soil sample (Heath *et al*., 2009). This esterase had no identity to any GenBank nucleotide sequence, and it is unlikely that it would have been detected by low stringency PCR-based screening methods or by deep sequencing techniques.

### Mining biocatalysts from extreme environments

The maximum yield of industrial processes is achieved by optimization of physicochemical parameters, and most currently available enzymes are incompatible with these conditions, and the use of enzymes requires that these processes be adapted, which can result in reduced production levels. Extreme environments, such as solfataric hot springs (Rhee *et al*., 2005), Urania hypersaline basins (Ferrer *et al*., 2005a), acid mine drainage biofilms (Guazzaroni *et al*., 2013), glacier soil (Yuhong *et al*., 2009), glacial ice (Simon *et al*., 2009) and Antarctic soil (Cieslinski *et al*., 2009; Heath *et al*., 2009) represent a rich and largely unexploited reservoirs of novel biocatalysts with biotechnologically valuable properties. Although the diversity of microbial communities present in many extreme habitats is likely to be low, samples from these environments are still a valuable source of novel enzymes that are active under extreme conditions (Steele *et al*., 2009), and as might be expected, the properties of the enzymes retrieved are consistent with conditions of the source environments (Feng *et al*., 2007; Heath *et al*., 2009; Jiang *et al*., 2009; Hu *et al*., 2012).

Metagenomic libraries derived from extreme habitats have been constructed and enzymes displaying environment compatible properties have been recovered. A relevant consideration is the compatibility of the host organism growth with the optimum physicochemical characteristics used in the screening of the catalytic activity. *Escherichia coli* is highly sensitive to the conditions that are typically present in extreme environments, and experimental conditions should therefore be adjusted to perform library screening under the conditions of interest (for example, low pH) while still allowing growth of this organism. Alternatively, screening could be performed without subjecting the host to any selective pressure, for example in cases where the host organism is used to clone total metagenomic DNA and the screening is performed under regular growth conditions. In both cases, after the initial identification of the enzymes, characterization of the desired catalytic properties of the source clones can be made with crude culture extracts and selected enzymes may then be purified for a more detailed characterization. For instance, a novel alkaliphilic esterase active at 7°C was identified by screening a metagenomic library from Antarctic desert soil (Heath *et al*., 2009). In a separate study, another cold-active esterase was also isolated from the same library using a similar approach (Hu *et al*., 2012). Their low-temperature activity and alkaliphilic properties make these enzymes interesting candidates for industrial applications. Esterases are of particular industrial interest since they serve as useful biocatalysts in the detergent and food industries, and for the production of fine chemicals and in bioremediation processes (Aurilia *et al*., 2008). Cold-adapted esterolytic enzymes could be of further value with regard to savings in energy, as compared with their mesophilic counterparts, due to their high catalytic activities at low temperatures (Hu *et al*., 2012). At the opposite extreme, Rhee and colleagues (2005) constructed fosmid libraries using environmental samples from solfataric hot springs, and function-based screening identified a novel esterase that exhibited a high temperature optimum activity and high thermal stability.

Cellulases are enzymes with a wide range of applications such as in the textile industry for cotton softening and denim finishing; in the food industry for mashing; and in the cellulose pulp and paper industries for de-inking, fiber refining and fiber modification (Bhat, 2000). Recent interest has focussed on the use of cellulases in the hydrolysis of pretreated lignocellulosic materials into sugars, which can then be fermented to produce second-generation ethanol and other bio-products. Metagenomic approaches have been widely employed to isolate cellulases from environmental samples in which lignocellulosic materials were under intensive degradation, including soil (Kim *et al*., 2008; Jiang *et al*., 2009), hindgut contents of higher termites (Warnecke *et al*., 2007), compost (Pang *et al*., 2009), contents of rabbit cecum (Feng *et al*., 2007), fresh cast from earthworms (Beloqui *et al*., 2010), Brazilian mangroves (Thompson *et al*., 2013), calf rumen (Ferrer *et al*., 2012) and cow rumen (Ferrer *et al*., 2005b; Wang *et al*., 2013). As anticipated, most cellulases retrieved from uncultured microbes from the rumen of herbivores were acidic and mesophilic, properties which are compatible with the conditions of yeast fermentation for the production of second-generation ethanol. Although a number of cellulase active clones have been isolated from metagenomic libraries by functional screening, the frequencies of true positive identifications were low due to low and biased expression of the majority of cellulase genes in *E. coli*. Moreover, most of the enzymes identified were endoglucanases (despite the use of specific substrates for screening other types of cellulases), which was probably a consequence of the expression of the correctly folded enzymes in *E. coli*. No cellobiohydrolases active against crystalline cellulose substrates have as yet been isolated from metagenomic libraries (Duan and Feng, 2010), which may be due to the fungal source of the majority of cellobiohydrolases and, to date, there are no reports using activity-based screening of cellulases from a cDNA metagenomic library in fungi. Moreover, since extreme environments are colonized mainly by microorganisms highly adapted to such harsh conditions, the majority of species are likely to be phylogenetically distant from *E. coli*. The recurring limitation of the metagenomic approach for certain enzyme families is therefore the lack of genetic tools for library screening in phylogenetically diverse hosts, and the development of such tools would significantly increase the probability of successful enzyme retrieval from a wide range of environmental samples.

### Using synthetic biology to improve metagenomic screening strategies

**Fig 2:**
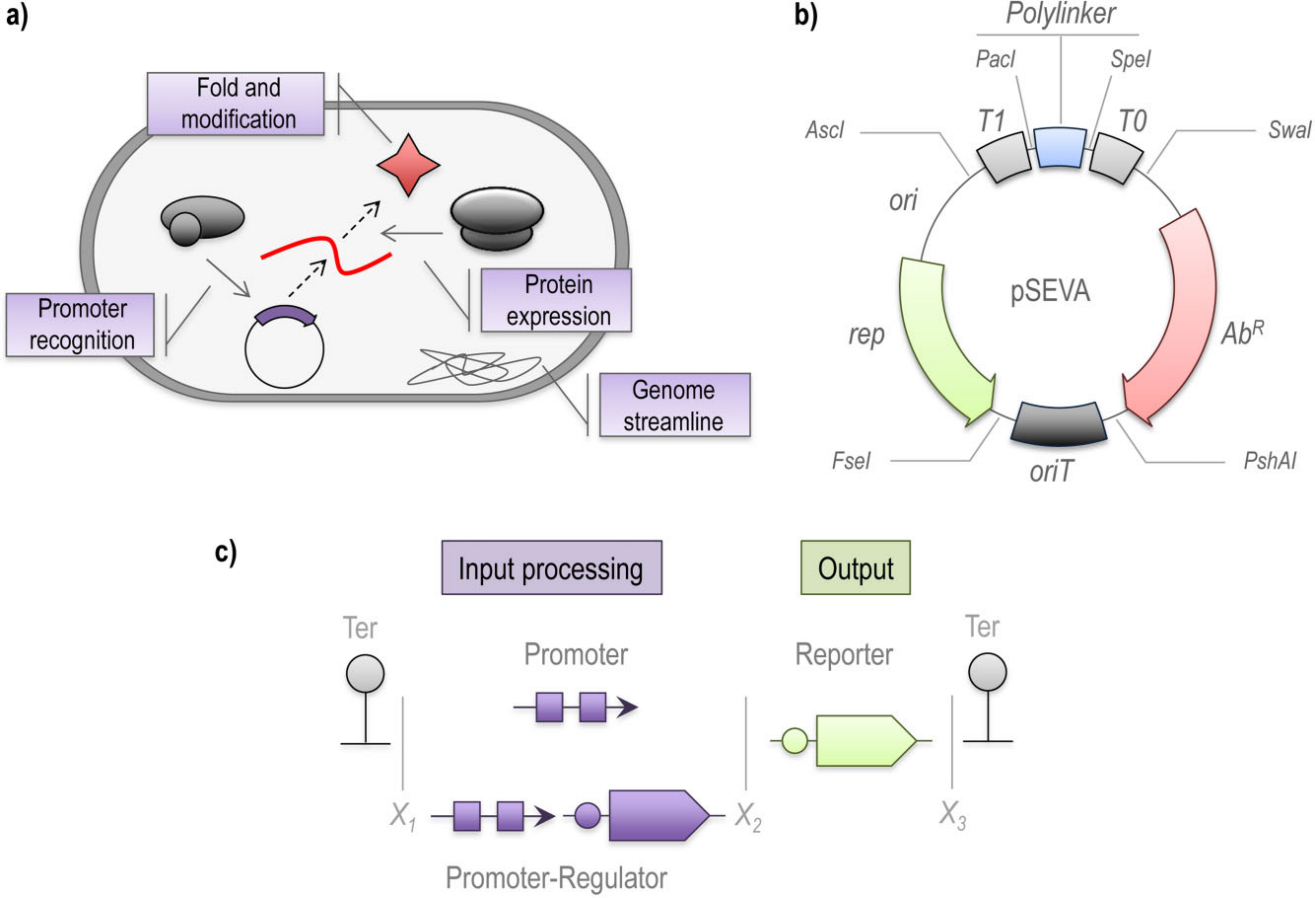
Synthetic Biology may overcome limiting steps in activity-based metagenomic library screening. Current bottlenecks in functional metagenomics are related to (A) limitations in the host capabilities, (B) the performance of the genetic tools and (C) the availability of efficient screening methods.A. In the case of the host, critical steps related to the recognition of transcriptional and translational signals, as well as the folding and modification of the expressed enzyme need to be enhanced. Host performance might be improved by reducing the metabolic burden related to the expression of unnecessary genes.B. The use of semi-synthetic, high-efficiency genetic tools is essential for the construction of metagenomic libraries that can be maintained and screened in a wide number of microorganisms. The example shows the pSEVA bacterial vector, which is endowed with several functional features such as terminators, origin for transfer and an extensive polylinker optimized for use in several bacterial hosts.C. Genetic circuits constructed by combining input modules (e.g. promoters and regulators) and output devices (such as reporter proteins) assembled with a standard format that uses the same sets of restriction enzymes (represented by X_1_, X_2_, etc.). Such circuits facilitate the screening of enzymatic activities in metagenomic libraries. The standardization of the assembly process facilitates the combination of several independent modules to construct sophisticated activity-trigged biosensors.

The previous sections have highlighted a number of bottlenecks facing metagenomic screening that need to be resolved in order to improve the discovery rate of target enzymes. These limitations can be grouped in three main categories (as shown in Fig. 2). First, there is a need for improvement in host organism capabilities with the aim of improving the expression of the target enzymes. Second, the development of new genetic tools is necessary in order to improve the construction of metagenomic libraries suitable for screening in different hosts. Finally, continuation of ongoing research to elaborate novel screening strategies that enhance the discovery rate of the enzymes of interest is needed. The advances in synthetic biology over the past decade could provide the framework to address these constrains, and a particularly promising approach is the analysis of biological systems in an analogous way as electronic devices, whereby cells can be reprogrammed to perform new tasks with high efficiency (Purnick and Weiss, 2009; Weber and Fussenegger, 2010). Synthetic biology relies on a conceptual framework more closely related to engineering than biology, such as design, modelling, implementation and debugging (Canton *et al*., 2008; Purnick and Weiss, 2009; Weber and Fussenegger, 2010). The design aspect focuses on the planning and construction of new gene circuits for the desired application (Canton *et al*., 2008). Modelling involves computational simulation of the proposed gene circuits in order to both evaluate performance and capabilities and to guide the selection of the suitable molecular components necessary for its construction (Koide *et al*., 2009). The implementation step encompasses the physical assembly of the DNA elements encoding the appropriate components (such as promoters, regulators, terminators, enzymes, transporters, etc.), and follows a specific assembly standard (Arkin, 2008). Finally, the debugging step requires the testing and validation of the circuit *in vivo*, and includes the correction of undesirable traits that have their origin in the emergent properties of biology systems (Gardner *et al*., 2000; Moon *et al*., 2011; Siuti *et al*., 2013). Several examples of new biological circuits that have been successfully designed and implemented are currently available (Gardner *et al*., 2000; Cox *et al*., 2007; 2010; Moon *et al*., 2011; Silva-Rocha and de Lorenzo, 2011), and in recent years, the field has developed at a remarkable speed (Weber and Fussenegger, 2010; Zhan *et al*., 2010; Regot *et al*., 2011; Siuti *et al*., 2013).

As mentioned in previous sections, failure of heterologous gene expression in host cells is among the main causes of the low recovery rates of enzymes of interest from metagenomic libraries (Handelsman, 2004; Lorenz and Eck, 2005). A combination of different strategies could be applied to optimize this critical event, (Fig. 2A). In the initial transcription step, a reduced affinity of the RNA polymerase (RNAP) for intrinsic promoters derived from metagenomic fragments represents an important limitation for heterologous protein expression in the host organism. Promoter recognition in prokaryotes is strongly biased among the different phylogenetic groups (Gabor *et al*., 2004), and the ideal host for heterologous protein expression should be endowed with transcriptional machinery with broad promoter recognition capability. Such a goal could be attained, for example, by coexpressing heterologous sigma factors with different promoter specificities (Osterberg *et al*., 2011; Rhodius *et al*., 2013), thereby permitting protein expression from promoters derived from different bacterial sources. In a more direct approach, the expression of foreign genes in the host organism could be driven by a high-efficiency expression system such as the T7 RNAP (Terron-Gonzalez *et al*., 2013), and has the advantage that genes controlled by complex signal transduction mechanisms could be easily expressed in response to a single inducer such as isopropyl-beta-D-thiogalactopyranoside (Tabor, 2001). The same line of reasoning may also be applied to mRNA translation, where poor recognition of the ribosome-binding site (RBS) can reduce protein expression levels (Zelcbuch *et al*., 2013). In this case, the coexpression of additional proteins related to the mRNA recognition step could expand the host capability for foreign gene expression (Uchiyama and Miyazaki, 2009). While these strategies aim to improve the level of expression of target proteins, an additional factor is related to the activity levels of expressed enzymes. This is particularly true in the case of enzymes displaying either complex folding or requiring additional processing steps (such as cleavage, secretion, or peptide modification) (DeSantis and Jones, 1999; Bhat, 2000). For instance, in the case of protein folding, the coexpression of molecular chaperons (Dobson, 2003) has been reported to enhance the expression of heterologous proteins in *E. coli* (Ferrer *et al*., 2004).

Among the additional approaches to improve host organism capabilities, genome edition (or streamlining) (Cambray *et al*., 2011) deserves special attention. Since many of the genes in bacteria such as *E. coli* are not essential for growth under standard laboratory conditions (Medini *et al*., 2005), maintaining these genes and the consequent redundant expression of unnecessary proteins represents a significant energetic cost and a metabolic burden to the host cell (Posfai *et al*., 2006). The reduction of genome size in bacteria by the removal of non-essential genes has been shown to endow bacteria with renewed metabolic vigor that enhances the production level of heterologous proteins (Posfai *et al*., 2006; Martinez-Garcia *et al*., 2014). Due to their superior expression capabilities, bacterial strains with minimized genomes are therefore promising host organisms for screening of metagenomic libraries.

In addition to genetic engineering of the more common hosts such as *E. coli*, several attempts have been focused on the use of alternative hosts to increase the rate of enzyme identification in metagenomic screening (Craig *et al*., 2010; Guazzaroni *et al*., 2013). Screening in alternative hosts requires either library construction in non-optimal, broad-host-range vectors (Craig *et al*., 2010) or subcloning of target genes in appropriate vectors (Guazzaroni *et al*., 2013). Universal and standardized tools are required in order to facilitate this type of multi-organism approach for the screening of metagenomic libraries in a larger number of hosts, and synthetic biology approaches are particularly suited to the development and use of such tools for the construction of adequate gene circuits in an increasing number of host cell platforms (Andrianantoandro *et al*., 2006; Arkin, 2008; Shetty *et al*., 2008). Among these new multihost tools, the pSEVA vectors are of particular interest as synthetic broad-host-range vectors that are expected to work in about 100 different bacterial species (Fig. 2B; Silva-Rocha *et al*., 2013). Using this system as a starting point, new genetic tools could be developed for cloning and screening of environmental DNA from phylogenetically diverse bacteria.

Currently available methods that are used to screen for new catalytic activities in metagenomic libraries frequently rely either on the use of chromogenic enzyme substrates (Hu *et al*., 2012; Ko *et al*., 2013) or substrates that when degraded leave a clear halo around positive colonies (Wong *et al*., 2009). More labor-intensive screening procedures such as colony PCR are also used for enzyme discovery (Hrvatin and Piel, 2007). The rapid expansion of synthetic biology has already resulted in the construction of regulatory circuits using well-characterized parts (Voigt, 2006), and there is a tremendous potential to use this accumulated knowledge for further advances in the design of biosensors to screen for enzymatic activities (Williamson *et al*., 2005; Nasuno *et al*., 2012). The use of biosensors is an *in vivo* strategy that has been used to identify specific enzymatic activities using engineered regulatory circuits coupled to a reporter gene, such as *lacZ*, green fluorescent protein or luciferase (Mohn *et al*., 2006; de Las Heras *et al*., 2010). This genetic trap approach (Uchiyama *et al*., 2005; Uchiyama and Watanabe, 2008; Uchiyama and Miyazaki, 2010) eliminates the necessity for extensive manipulation during screening, yet allows the identification of positive clones in metagenomic libraries. Synthetic biology approaches have also been used to modulate enzyme levels in biosynthetic pathways by combinatorially pairing genes with a defined set of RBS (Zelcbuch *et al*., 2013), resulting in the modulation of protein abundance by several orders of magnitude, which showed that engineering of metabolic pathways relies on precise control of enzyme levels (Zelcbuch *et al*., 2013). These examples demonstrate how synthetic biology approaches can improve the ability to interconnect regulatory components (e.g. promoters and regulators) to generate new circuits with reliable performance characteristics (Fig. 2C; Schmidt and de Lorenzo, 2012). The combination of the available assembly platforms for circuit engineering (Arkin, 2008; Zhan *et al*., 2010; Nikel and de Lorenzo, 2013) together with approaches for the redesign of regulatory systems to recognize new molecules (de Las Heras and de Lorenzo, 2012) is likely to lead to new concepts and allow the implementation of new genetic traps for the identification of enzymatic activities of interest (Fernandez-Arrojo *et al*., 2010), and will have a significant impact in the detection rate of new biotechnologically relevant biocatalysts (Ferrer *et al*., 2009).

## Concluding remarks

Classic approaches for isolating industrial enzymes involve their identification from cultured microorganisms, and more recently, this strategy has been expanded to include *in vitro* evolution and rational design techniques for the improvement of their catalytic properties. However, the diversity of proteins identified using this strategy is restricted, the methods are time-consuming, costly and in the case of *in vitro* evolution, it is impossible to test all variants (Sommer *et al*., 2010). The metagenomics approach encompasses the idea that the desired biocatalyst may already exist in nature, and is an alternative strategy that is used to explore the inherent diversity of natural environments. As a result, development of a broad repertoire of culture-independent techniques have been developed and advances towards the identification of novel and potent biocatalysts from metagenomic libraries have been made. However, the available metagenomic approaches require further refinement to achieve the goal of identifying industrially relevant biocatalysts. Existing limitations with respect to host expression, vector availability and specific screening restrictions cannot be solved by using a single approach, and requires the synergic implementation of multiple methodologies. A growing number of studies have shown that synthetic biology may significantly improve metagenomic library screening and allow exploitation of the rich biochemical potential present in natural environments.

## Conflict of Interest

None declared.
